# Isolated thoracic intramedullary Erdheim-Chester disease presenting with paraplegia: a case report and literature review

**DOI:** 10.1186/s12891-021-04061-7

**Published:** 2021-03-12

**Authors:** Ikchan Jeon, Joon Hyuk Choi

**Affiliations:** 1 Department of Neurosurgery, Yeungnam University Hospital, Yeungnam University College of Medicine, Hyeonchung street 170, 42415 Daegu, South Korea; 2 Department of Pathology, Yeungnam University Hospital, Yeungnam University College of Medicine, Daegu, South Korea

**Keywords:** Erdheim‐chester disease, Histiocytosis, Spinal cord, Intramedullary, Tumor

## Abstract

**Background:**

Erdheim-Chester disease (ECD) is a rare, idiopathic, systemic non-Langerhans cell histiocytosis involving long bone and visceral organs. Central nervous system (CNS) involvement is uncommon and most cases develop as a part of systemic disease. We present a rare case of variant ECD as an isolated intramedullary tumor.

**Case presentation:**

A 75-year-old female patient with a medical history of diabetes and hypertension presented with sudden-onset flaccid paraparesis for 1 day. Neurological examination revealed grade 2–3 weakness in both legs, decreased deep tendon reflex, loss of anal tone, and numbness below T4. Leg weakness deteriorated to G1 before surgery. Preoperative magnetic resonance imaging (MRI) and ^18^F-fluorodeoxyglucose positron emission tomography/computed tomography (FDG-PET/CT) showed an intramedullary mass lesion at T2-T4 with no systemic lesion, which was heterogeneous enhancement pattern with cord swelling and edema from C7 to T6. Gross total removal was achieved for the white-gray-colored and soft-natured intramedullary mass lesion with an ill-defined boundary. Histological finding revealed benign histiocytic proliferation with foamy histiocytes and uniform nuclei. We concluded it as an isolated intramedullary ECD. The patient showed self-standing and walkable at 18-month with no evidence of recurrence and new lesion on spine MRI and whole-body FDG-PET/CT until sudden occurrence of unknown originated thoracic cord infarction.

**Conclusions:**

We experienced an extremely rare case of isolated intramedullary ECD, which was controlled by surgical resection with no adjuvant therapy. Histological examination is the most important for final diagnosis, and careful serial follow-up after surgical resection is required to identify the recurrence and progression to systemic disease.

## Background

Erdheim-Chester disease (ECD) is a rare, idiopathic, systemic non-Langerhans cell histiocytosis (LCH). It is characterized by foamy histiocytes surrounded by fibrosis, and mainly affects middle-aged adults [1, 2]. The main clinical feature of ECD is the pain secondary to bilateral osteosclerosis of the long bones. Skeletal involvement occurs in more than 95 % of ECD cases. Extraskeletal manifestations can occur in the lung, heart, skin, kidney, retroperitoneum, and orbit [3–8]. Central nervous system (CNS) involvement is uncommon, and most cases develop as intracranial lesions, which are identified as a part of the systemic disease [1, 9, 10]. Isolated cases of ECD development in the CNS are extremely rare, and only two cases of the isolated occurrence of intracranial ECD have been reported previously [3, 11]. To our knowledge, no isolated case of ECD developing as a spinal intramedullary tumor has been reported. Herein, we report the diagnosis, surgical treatment, and 1-year follow-up for a case of isolated thoracic intramedullary ECD presenting as sudden paraplegia.

## Case presentation

**Fig. 1:**
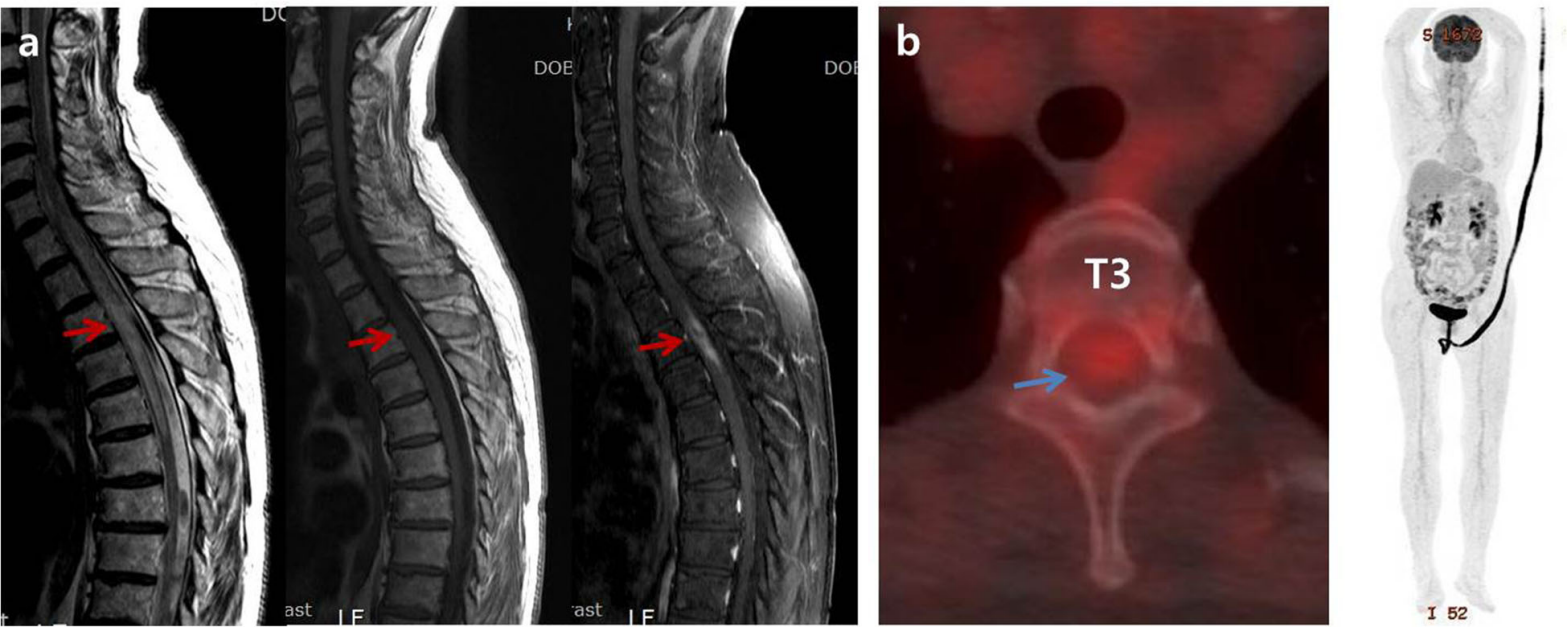
Preoperative magnetic resonance imaging shows intramedullary mass lesion with iso-intense on T2-weighted images (WI) and iso- to slightly low-intense on T1-WI on T2-4, which was heterogeneous enhanced pattern with cord swelling and edema from C7 to T6 (red arrows) (**a**). There was a single lesion on T2-4 with maximum standardized uptake value (SUV_max_) of 3.14 and no systemic lesion on whole-body ^18^F-fluorodeoxyglucose positron emission tomography/computed tomography (blue arrow) (**b**)

A 75-year-old female patient presented with sudden-onset flaccid paraparesis for 1 day. The patient had a history of diabetes and hypertension. Neurological examination showed grade 2–3 motor weakness of both legs, decreased deep tendon reflex, loss of anal tone, and numbness below the T4 dermatome. There was no fever, cardiopulmonary symptoms, pain of extremities, or palpable lymph nodes. Magnetic resonance imaging (MRI) revealed an intramedullary mass lesion on T2-T4 with cord swelling and edema from C7 to T6. The lesion was iso-intense on T2-weighted images (WI) and iso- to slightly low-intense on T1-WI with heterogeneous enhancement (Fig. 1a). No additional lesions were found on the brain and whole-spine MRI. Whole-body ^18^F-fluorodeoxyglucose positron emission tomography/computed tomography (FDG-PET/CT) confirmed the single lesion on T2-4 with a maximum standardized uptake value (SUV_max_) of 3.14 and no systemic lesion (Fig. 1b). Leg weakness deteriorated to G1 prior to surgery.

**Fig. 2:**
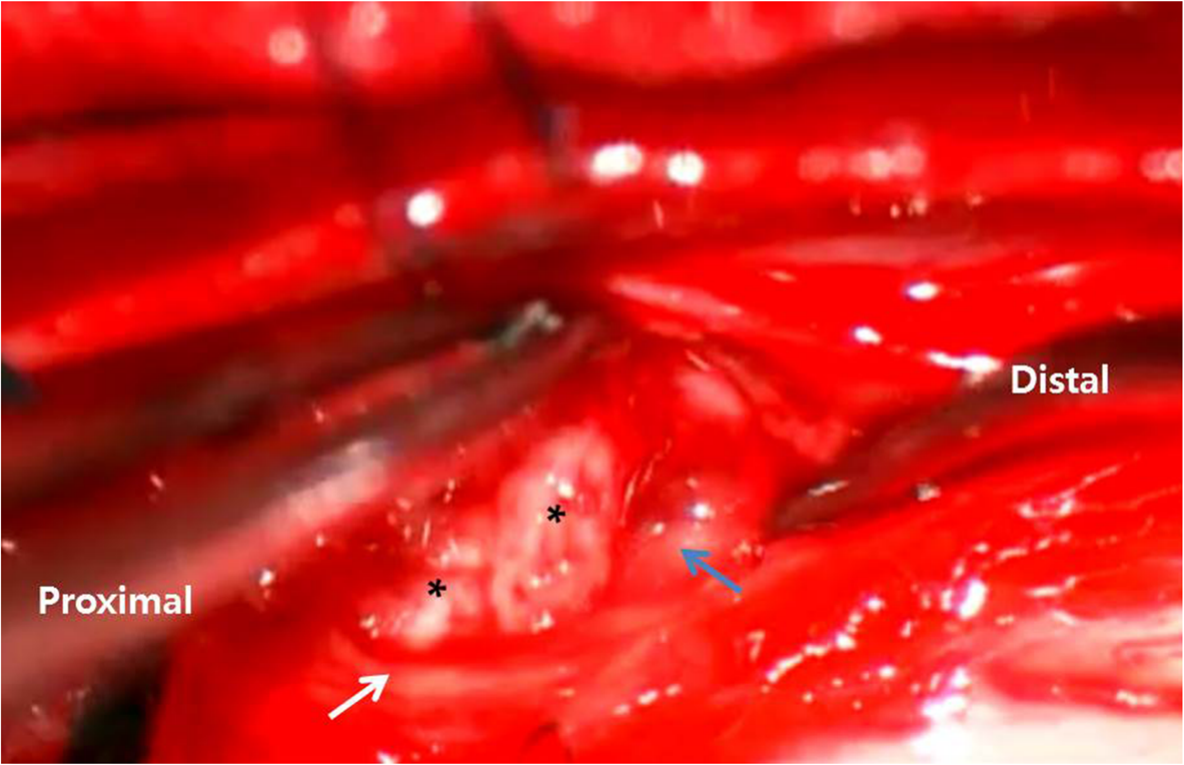
Intraoperative findings. White-gray-colored and soft-natured mass lesion (black asterisks) with ill-defined boundary is detaching from the inside surface of thoracic cord (blue arrow) under midline myelotomy with opening pia mater (white arrow)

**Fig. 3:**
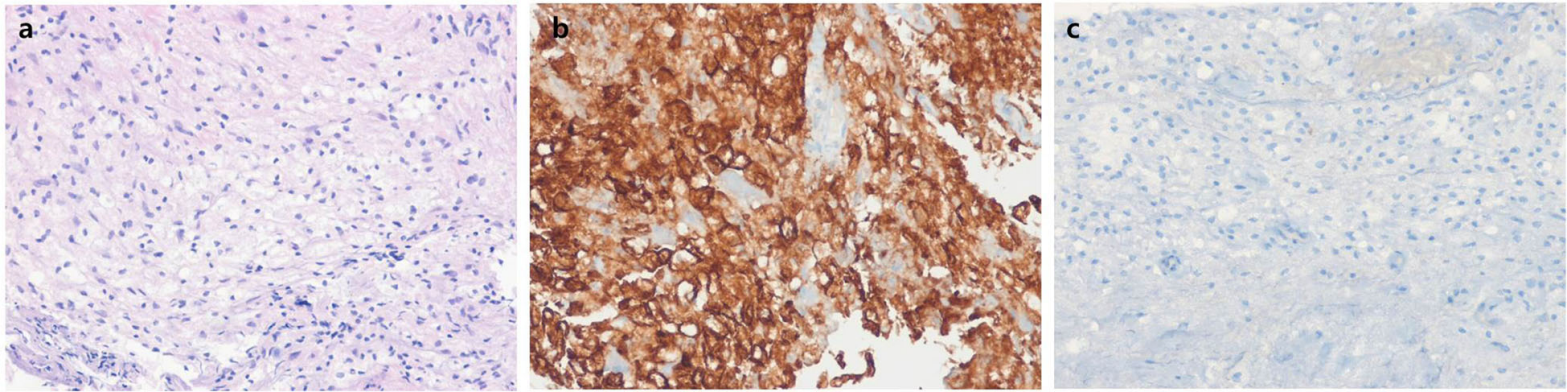
Histological findings. The lesion shows aggregates of foamy (lipid-laden) histiocytes with uniform nuclei and abundant cytoplasm (hematoxylin-eosin stain, original magnification × 200) (**a**). Foamy histiocytes show strong and diffuse expression of CD163 (immunohistochemical stain for CD163, original magnification × 200) (**b**), and negative for CD1a (immunohistochemical stain for CD1a, original magnification × 400) (**c**)

**Fig. 4:**
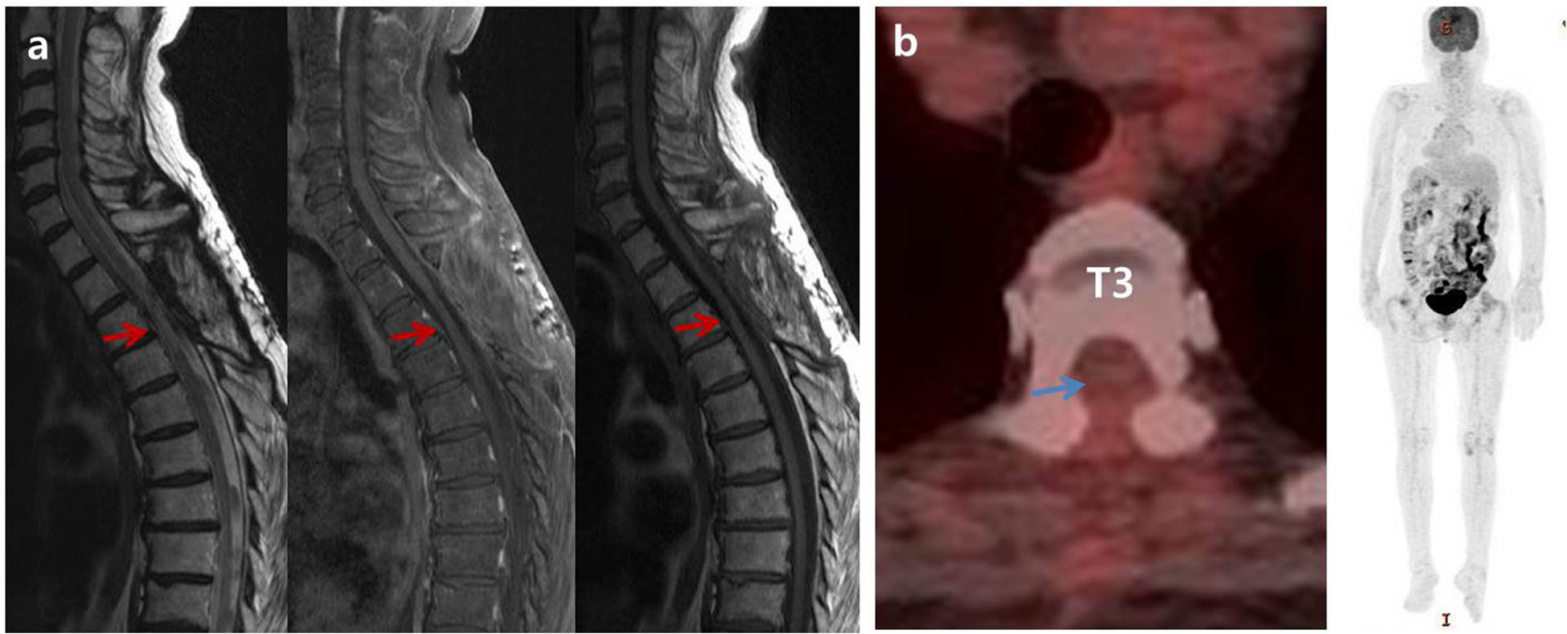
There was no new lesions or recurrence (red and blue arrows) on magnetic resonance imaging (**a**) and whole-body ^18^F-fluorodeoxyglucose positron emission tomography/computed tomography (**b**) at 1-year follow-up

**Fig. 5:**
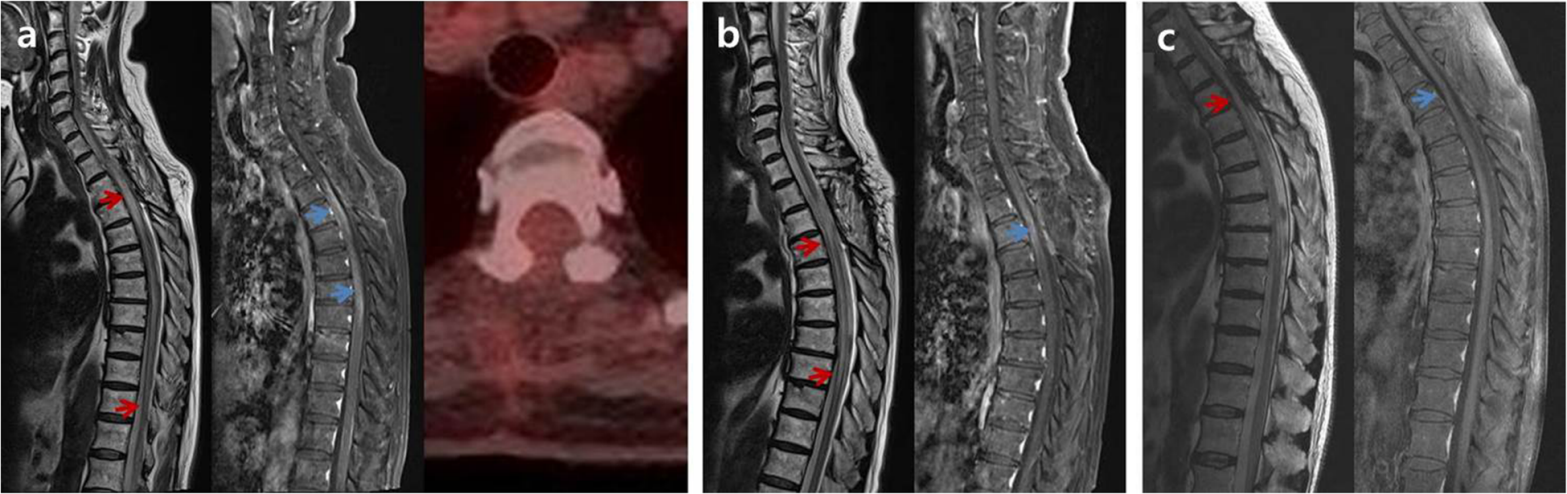
On the initial radiological findings after development of paraplegia at 18-month follow-up (**a**), magnetic resonance imaging (MRI) showed swelling, extensive edema (red arrows), and contrast enhancement (blue arrows) of whole thoracic cord including previous tumor surgical lesion on T2-4. However, there is no evidence of recurrence or new lesion on whole-body ^18^F-fluorodeoxyglucose positron emission tomography/computed tomography. On the 2-week follow-up MRI, there was significant reduction of edema and contrast enhancement in thoracic cord (red and blue arrows) (**b**), which was almost disappeared on the 5-week follow-up MRI (**c**)

Under the laminectomy of T2-T4 and opening the dura, enlarged thoracic cord with a normal superficial appearance was noted. Midline myelotomy allowed the identification of a white-gray-colored and soft-natured mass lesion with an ill-defined boundary (Fig. 2). The mass lesion was located at the central portion with a slight deviation on the right inside the cord. Gross total removal was achieved under intraoperative neuro-monitoring with no signal change. The patient showed no change in neurologic function after surgery. Histological examination revealed diffuse infiltration of foamy (lipid-laden) histiocytes with uniform nuclei and abundant cytoplasm (Fig. 3a). On immunohistochemical staining, foamy histiocytes showed diffuse and strong expression of CD163 and CD68 (histiocytic marker) (Fig. 3b), and there were negative for CD1a, S100, and BRAF^V600E^ mutation (Fig. 3c). No systemic lesions were noted on the preoperative radiological examinations; therefore, we confirmed this case as variant ECD with isolated intramedullary involvement. There was no recurrence or new development of mass lesions on MRI and whole-body FDG-PET/CT at 12-month follow-up (Fig. 4). The patient became self-standing and aid-walkable with recovery of anal tone and sensory impairments. Unfortunately, sudden paraplegia occurred again at 18-month follow-up, which was confirmed that it was developed by unknown originated thoracic cord infarction. There was no evidence related with recurrence or new lesion of ECD on spine MRI and whole-body FDG-PET/CT (Fig. 5).

## Discussion and Conclusions

Non-LCH in the CNS include juvenile xanthogranuloma (JXG), adult-onset xanthogranuloma, xanthoma disseminatum, Rosai-Dorfman disease (RDD), and ECD [12]. It can be difficult to distinguish among these entities as there are significant overlaps in their clinicopathological characteristics [3]. Preoperative imaging and laboratory studies do not differentiate ECD from these diseases; therefore, histological confirmation is the absolute method for a final diagnosis. CNS involvement has been reported in up to 50 % of ECD cases [13–15]. It commonly affects neurohypophysis causing diabetes insipidus and can be also located in the brainstem, cerebellum, middle cerebellar peduncle, cerebellar hemisphere, and basal ganglia [9, 10]. Spinal cord involvement may also occur due to either extramedullary masses or intra-axial infiltration as a part of the extraskeletal lesions under systemic involvement [9, 16, 17]. Most cases of spinal involvement showed as subdural or epidural lesion regardless of systemic disease. Two cases of intramedullary spinal cord lesions with systemic involvement and two cases of isolated spinal ECD with subdural and epidural masses causing spinal cord compression have been reported previously [16, 18, 19].

Primary or metastatic spinal cord tumors, multiple sclerosis, and sarcoidosis should be considered as differential diagnoses [16]. Old age, rapid progression of symptoms, intrameduallly lesion with cord edema, ring-enhanced pattern, and no systemic lesion on FDG-PET in this patient, led to difficulties in differentiating ECD from other intramedullary lesions before pathological confirmation. The initial preoperative diagnosis was high-grade glioma. Non-surgical treatments, such as radiotheraphy, were considered due to the old age and prognosis of malignant lesion. However, we identified relatively low FDG-uptake (3.14 of SUV_max_) of the intramedullary lesion when compared with the higher FDG-uptake seen with malignant lesions. Naito et al. [20] demonstrated significant accumulation of FDG in a spinal intramedullary tumor classified as high-grade malignancy. In addition, the SUV_max_ was higher in CNS lesions of eight ECD patients (5.1–16) than in this patient [21]. These patients were diagnosed as having ECD with systemic involvements, and all CNS involvements were brain lesions, with no spinal cord involvement. We think that isolated type and spinal cord involvement in ECD can show different disease activity and SUV uptake, and additional study is required with more data.

In this case, we identified the patient was negative for BRAF^V600E^ mutation, although there were foamy histiocytes with CD68 (+) and CD1a (-). Haroche et al. [22] reported that BRAF^V600E^ mutation was detected in 54 % (13/24) of ECD and 38 % (11/29) of LCH, which might benefit from targeted therapy. Negative BRAF^V600E^ mutation in ECD is required to differentiate with RDD and JXG, which are also negative for BRAF^V600E^ mutation [23]. However, in this case, there was no emperipolesis, which is the characteristic histological feature with extensive lymphadenopathy of RDD in the histological examination. Moreover, our patient was too old to be considered as having JXG, which is a benign pediatric histiocytosis and resolved spontaneously. In particular, the rate of positive BRAF^V600E^ varies depending on the biopsy sites; there was only 13 % (6/46) of positive BRAF^V600E^ in CNS [22]. Considering abovementioned features of BRAF^V600E^, we cannot exclude the possibility of ECD because negative BRAF^V600E^ is not identified. Rather, the histological findings are suitable for the confirmation of ECD.

The prognosis of ECD appears to be significantly worse than that of other types of histiocytosis, which is owing to the extent of visceral involvement. A progressive course is commonly associated with a high mortality rate, which is found to be 22 % in the literature [4, 9, 24]. The main causes of death are lung fibrosis, leading to respiratory and cardiac failures, or renal failure secondary to retroperitoneal fibrosis [4, 25]. Unfortunately, there is no consensus on the treatment due to the small number of case reports or series that do not include long follow-up periods because of its low incidence. Corticosteroids, radiation therapy, chemotherapy, immunotherapy, bisphosphonate, surgery, or a combination of these therapies have been suggested [3, 11]. Recently, pegylated-interferon-alpha (PEG-INF-α), INF-α, and anakinra have been commonly used as first-line therapies. Various second-line agents include cladribine, imatinib, and infliximab [26]. However, there are insufficient data on the effectivity and side effects of these treatments.

Histiocytosis classification has been revised recently with the following five categories: (1) Langerhans-related, (2) cutaneous and mucocutaneous, (3) malignant histiocytosis, (4) Rosai-Dorfman disease, and (5) hemophagocytic lymphohistiocytosis and macrophage activation syndrome [27]. ECD is classified as Langerhans-related group with LCH and extracutaneous JXG among the five categories. ECD is theoretically considered a progressive disease with gradual involvement of multiple organ systems, which can produce a wide spectrum of clinical manifestations. Therefore, early diagnosis and disease-modulating treatment are recommended to improve prognosis and survival [15]. There were no new lesions or recurrence at 1-year follow-up period after gross total removal of isolated intramedullary ECD lesion without adjuvant therapy. However, additional serial follow-up after surgical resection is required to identify recurrence and progression to systemic disease. Further studies of a large number of patients with isolated ECD are required to demonstrate the natural course of the disease and identify appropriate treatment strategies in a long-term perspective.

We experienced an extremely rare case of isolated intramedullary ECD; there were no new lesions or recurrence at 1-year follow-up period after gross total removal of isolated intramedullary ECD lesion without adjuvant therapy. Histological examination is the most important for final diagnosis, and careful serial follow-up after surgical resection is required to identify recurrence and progression to systemic disease.

## Data Availability

The datasets during and/or analysed during the current study are available from the corresponding author on reasonable request.

## Abbreviations

ECD: Erdheim-Chester disease
LCH: non-Langerhans cell histiocytosis
CNS: Central nervous system
MRI: magnetic resonance imaging
FDG-PET/CT: ^18^F-fluorodeoxyglucose positron emission tomography/computed tomography
SUV_max_: maximum standardized uptake value

## References

[1] Adle-Biassette H, Chetritt J, Bergemer-Fouquet AM, Wechsler J, Mussini JM, Gray F (1997). Pathology of the central nervous system in Chester-Erdheim disease: report of three cases. J Neuropathol Exp Neurol.

[2] Brower AC, Worsham GF, Dudley AH (1984). Erdheim-Chester disease: a distinct lipoidosis or part of the spectrum of histiocytosis?. Radiology.

[3] Conley A, Manjila S, Guan H, Guthikonda M, Kupsky WJ, Mittal S (2010). Non-Langerhans cell histiocytosis with isolated CNS involvement: an unusual variant of Erdheim-Chester disease. Neuropathology.

[4] Veyssier-Belot C, Cacoub P, Caparros-Lefebvre D, Wechsler J, Brun B, Remy M (1996). Erdheim-Chester disease. Clinical and radiologic characteristics of 59 cases. Med (Baltim).

[5] Dickson BC, Pethe V, Chung CT, Howarth DJ, Bilbao JM, Fornasier VL (2008). Systemic Erdheim-Chester disease. Virchows Arch.

[6] Chung JH, Park MS, Shin DH, Choe KO, Kim SK, Chang J (2005). Pulmonary involvement in Erdheim-Chester disease. Respirology.

[7] Allen TC, Chevez-Barrios P, Shetlar DJ, Cagle PT (2004). Pulmonary and ophthalmic involvement with Erdheim-Chester disease: a case report and review of the literature. Arch Pathol Lab Med.

[8] Sheu SY, Wenzel RR, Kersting C, Merten R, Otterbach F, Schmid KW (2004). Erdheim-Chester disease: case report with multisystemic manifestations including testes, thyroid, and lymph nodes, and a review of literature. J Clin Pathol.

[9] Lachenal F, Cotton F, Desmurs-Clavel H, Haroche J, Taillia H, Magy N (2006). Neurological manifestations and neuroradiological presentation of Erdheim-Chester disease: report of 6 cases and systematic review of the literature. J Neurol.

[10] Brodkin CL, Wszolek ZK (2006). Neurologic presentation of Erdheim-Chester disease. Neurol Neurochir Pol.

[11] Rushing EJ, Bouffard JP, Neal CJ, Koeller K, Martin J, Ozdemirli M (2004). Erdheim-Chester disease mimicking a primary brain tumor. Case report. J Neurosurg.

[12] Zelger BW, Sidoroff A, Orchard G, Cerio R (1996). Non-Langerhans cell histiocytoses. A new unifying concept. Am J Dermatopathol.

[13] Cives M, Simone V, Rizzo FM, Dicuonzo F, Cristallo Lacalamita M, Ingravallo G (2015). Erdheim-Chester disease: a systematic review. Crit Rev Oncol Hematol.

[14] Mazor RD, Manevich-Mazor M, Shoenfeld Y (2013). Erdheim-Chester Disease: a comprehensive review of the literature. Orphanet J Rare Dis.

[15] Arnaud L, Hervier B, Neel A, Hamidou MA, Kahn JE, Wechsler B (2011). CNS involvement and treatment with interferon-alpha are independent prognostic factors in Erdheim-Chester disease: a multicenter survival analysis of 53 patients. Blood.

[16] Takeuchi T, Sato M, Sonomura T, Itakura T (2012). Erdheim-Chester disease associated with intramedullary spinal cord lesion. Br J Radiol.

[17] Curgunlu A, Karter Y, Ozturk A (2003). Erdheim-Chester disease: a rare cause of paraplegia. Eur J Intern Med.

[18] Albayram S, Kizilkilic O, Zulfikar Z, Islak C, Kocer N (2002). Spinal dural involvement in Erdheim-Chester disease: MRI findings. Neuroradiology.

[19] Tzoulis C, Gjerde IO, Softeland E, Neckelmann G, Strom E, Vintermyr OK (2012). Erdheim-Chester disease presenting with an intramedullary spinal cord lesion. J Neurol.

[20] Naito K, Yamagata T, Arima H, Abe J, Tsuyuguchi N, Ohata K (2015). Qualitative analysis of spinal intramedullary lesions using PET/CT. J Neurosurg Spine.

[21] Arnaud L, Malek Z, Archambaud F, Kas A, Toledano D, Drier A (2009). 18F-fluorodeoxyglucose-positron emission tomography scanning is more useful in followup than in the initial assessment of patients with Erdheim-Chester disease. Arthritis Rheum.

[22] Haroche J, Charlotte F, Arnaud L, von Deimling A, Helias-Rodzewicz Z, Hervier B (2012). High prevalence of BRAF V600E mutations in Erdheim-Chester disease but not in other non-Langerhans cell histiocytoses. Blood.

[23] Ozkaya N, Rosenblum MK, Durham BH, Pichardo JD, Abdel-Wahab O, Hameed MR (2018). The histopathology of Erdheim-Chester disease: a comprehensive review of a molecularly characterized cohort. Mod Pathol.

[24] Haroche J, Abla O (2015). Uncommon histiocytic disorders: Rosai-Dorfman, juvenile xanthogranuloma, and Erdheim-Chester disease. Hematology Am Soc Hematol Educ Program.

[25] Rush WL, Andriko JA, Galateau-Salle F, Brambilla E, Brambilla C, Ziany-bey I (2000). Pulmonary pathology of Erdheim-Chester disease. Mod Pathol.

[26] Diamond EL, Dagna L, Hyman DM, Cavalli G, Janku F, Estrada-Veras J (2014). Consensus guidelines for the diagnosis and clinical management of Erdheim-Chester disease. Blood.

[27] Emile JF, Abla O, Fraitag S, Horne A, Haroche J, Donadieu J (2016). Revised classification of histiocytoses and neoplasms of the macrophage-dendritic cell lineages. Blood.

